# Influence of Human–Computer Interaction-Based Intelligent Dancing Robot and Psychological Construct on Choreography

**DOI:** 10.3389/fnbot.2022.819550

**Published:** 2022-05-18

**Authors:** Liu Yang

**Affiliations:** ^1^College of Arts, Hunan University of Arts and Sciences, Changde, China; ^2^Faculty of Music and Performing Arts, Sultan Idris Education University, Tanjung Malin, Malaysia

**Keywords:** dance creation, Human–Computer Interaction, Artificial Intelligence, deep learning, dancing robot

## Abstract

To study the influence of Artificial Intelligence (AI) on dancing robots in choreography, this paper introduces the biped-humanoid robot-imagined choreography model alongside the Psychological Space Construction (Psychological Construct) and Human–Computer Interaction (HCI). The proposed model is based on deep learning and imitating human thinking and is capable of imagining new dance elements. Finally, simulation experiments are designed to verify the model's effectiveness. Dance professionals are invited to evaluate the robot-imagined dance posture. The results show that the proposed model can vividly imitate human dancers and imagine and create new dance movements. The average basic feature retention and innovation scores of 30 new dance elements imagined on the L_1_ (head) are 7.29 and 7.64, respectively. By comparison, similar scores on 30 new elements in L_2_ (upper-body) are 7.73 and 7.40, respectively. Therefore, the proposed intelligent robot-imagined choreography model can help the dancing robot choreograph more finely and improve the choreography efficiency. The research results have significant practical value for dance teaching.

## Introduction

Dance, as an art, conveys people's emotional and ideal aesthetics through the relationship between body and spirit (Li, 2020). In the art of choreography, to pursue a better theme, the artistic works must appeal to choreographers' thoughts and emotions through a vague mental state in the psychological space. From the perspective of art, the Psychological Space Construction (Psychological Construct) is mainly affected by personal life experience, inner emotional experience, and cognitive activities. Meanwhile, dance is one of the art categories closest to psychology and linguistics. It takes the body as the material medium and entirely plays the role of body language through imagination and technical skills and connects human emotions. Besides, the body and psychology relationship is one of the most basic but delicate relations. Body language can convey and visualize psychological information through specific actions, the basic body language unit. Every action stirs a response from an individual's cognition. The image mapping in the human mind based on instantaneous thinking is an imaginary experience. As art forms flourish and human cognition deepens, new elements are constantly being injected into the psychological space and thus, enrich the source of chorographical innovation (Lei and Rau, 2021). Biped robot research involves multidisciplinary, including machinery, electronics, automation, control technology, and bionics. It has made high achievements in mechatronics. In particular, the gait analysis of the biped dancing robot can provide technical support for human research and prosthetics development when constructing a stable biped gait model. Dancing robots can also arouse teenagers' curiosity and enthusiasm for learning new technology by showing a stable gait and planning sports in the entertainment industry.

The technical level of biped robot gait research is not mature. Its stable gait and balance ability are still the research focus. Control and planning the robot's gait so that the robot's walking motion is always stable is of great practical importance. Barnes et al. (2021) observed that using robots to treat children with autism spectrum was promising and aroused interest in the research community. Children could interact with robots, such as dancing with robots and improve their concentration abilities. Kobayashi et al. (2022) proposed an exploratory study based on impromptu dance to explore the interaction between human dancers and mobile robots. A basic improvisation action algorithm was developed after many iterations with professional dancers. The robot trained in different dance styles created three unique original performances. Hua et al. (2021) researched the dancing robot, inputted the video content of dancing, estimated the human posture in the video with the help of the deep learning method, and obtained the coordinates and positions of the main points of the human body. Although there are many studies on dancing robots, there is a lack of research on humanoid dancing robots that can choreograph independently. Accordingly, the present work presents a method to provide a new direction for robot choreography.

The main contribution is to study the impact of Artificial Intelligence (AI) on dancing robots in choreography by introducing robot choreography and Psychological Construct. Most of the existing research focuses on robot dance's action design and research direction. Compared with the existing research, the innovation lies in establishing a choreography model that enables a bipedal humanoid robot to choreograph by imitating human thinking actively. Being able to choreograph beautiful dance moves using robots opens up new ideas for future research on dance creation.

## Research Methods

### Robot Choreography and Psychological Construct

**Figure 1:**
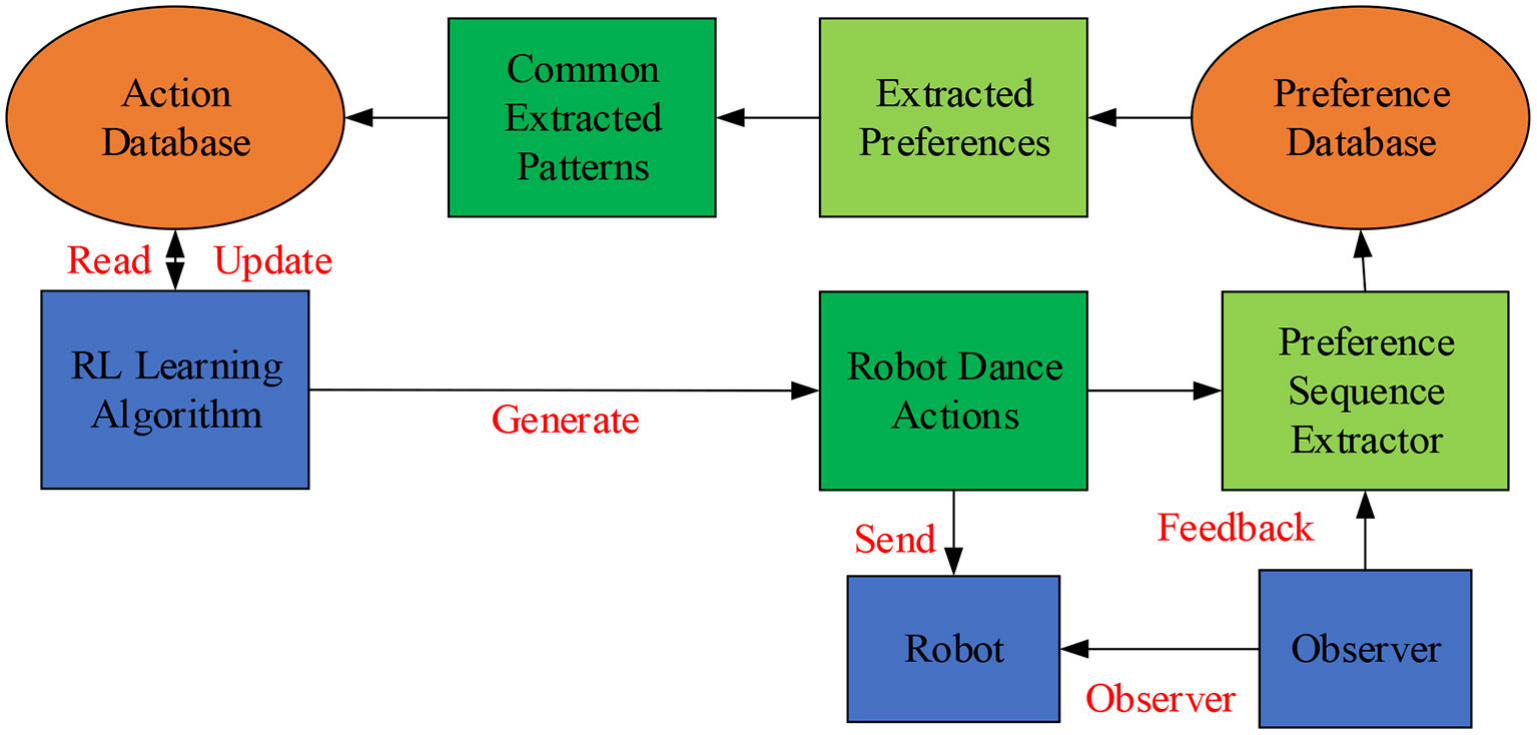
Principle of dancing robot-imagined choreography with enhanced interactive learning.

Dance experts often use familiar dance postures in choreography. Even similar dance postures exert different artistic tastes due to human dancers' individual charisma. Nevertheless, those uniformly programmed dancing robots' monotonous movements might easily get people disinterested after a few tryouts (Dong et al., 2021). Human–Computer Interaction (HCI) refers to the information exchange process between people and computers using a machine-understandable language to complete specific tasks in a certain interactive way. HCI studies the interactive relationship between system and user. The system can be machines or computerized systems and software. The HCI is realized through the external equipment with input/output functions or the corresponding software. Therefore, designers should give full play to the HCI of dancing robots to refine robot movements. The dancing robot-imagined choreography methods include random generation and mapping rules. In particular, the dancing robot-imagined model incorporates robot–human cooperation, dance posture learning, and robot-imagined choreography (Samosir and Widodo, 2020). Here, robot-imagined choreography refers to independently choreographing high-quality dance postures by the robot or through synchronized human–robot interaction. It is essentially the robot's intelligent behavior. High-quality dance can be defined explicitly through three features: maintaining the basic characteristics of human dance, innovation, and conforming to human aesthetics. The principle of robot-imagined interactive choreography is shown in Figure 1.

Figure 1 shows the choreography principle of the dancing robot with enhanced interactive learning. The system is constructed based on interactive Reinforcement Learning (RL). Then, by selecting dance actions with a high return, the robot will choreograph different dance moves considering human preferences. The Sarsa RL algorithm is used to update the dance action database in the choreograph system. Each action is given a unique cumulative return, and the Softmax action selection algorithm is used to select the appropriate dance actions to form dance works. At the same time, robots are encouraged to choose dance movements with higher returns over lower returns. Apparently, the Psychological Construct in choreography is a natural and intangible process of imaginative dance ideas. It is the psychological factory for processing dance works (Dou et al., 2021).

Personal experience, skills, cognition, and educational levels all contribute to artistic creation. However, without Psychological Construct, these elements are independent of each other and cannot lead to artic representations. Thus, material and spiritual (psychological) constructs are both essential for art. Like other art forms, choreography generates a series of psychological behaviors with complex interactions and relations, where the subject's imagination exerts the most active psychological function (Borovica, 2020). That is to say, a choreographer without artistic imagination can never choreograph aesthetic dance postures, nor can a positive and independent artistic dance image be formed.

**Figure 2:**
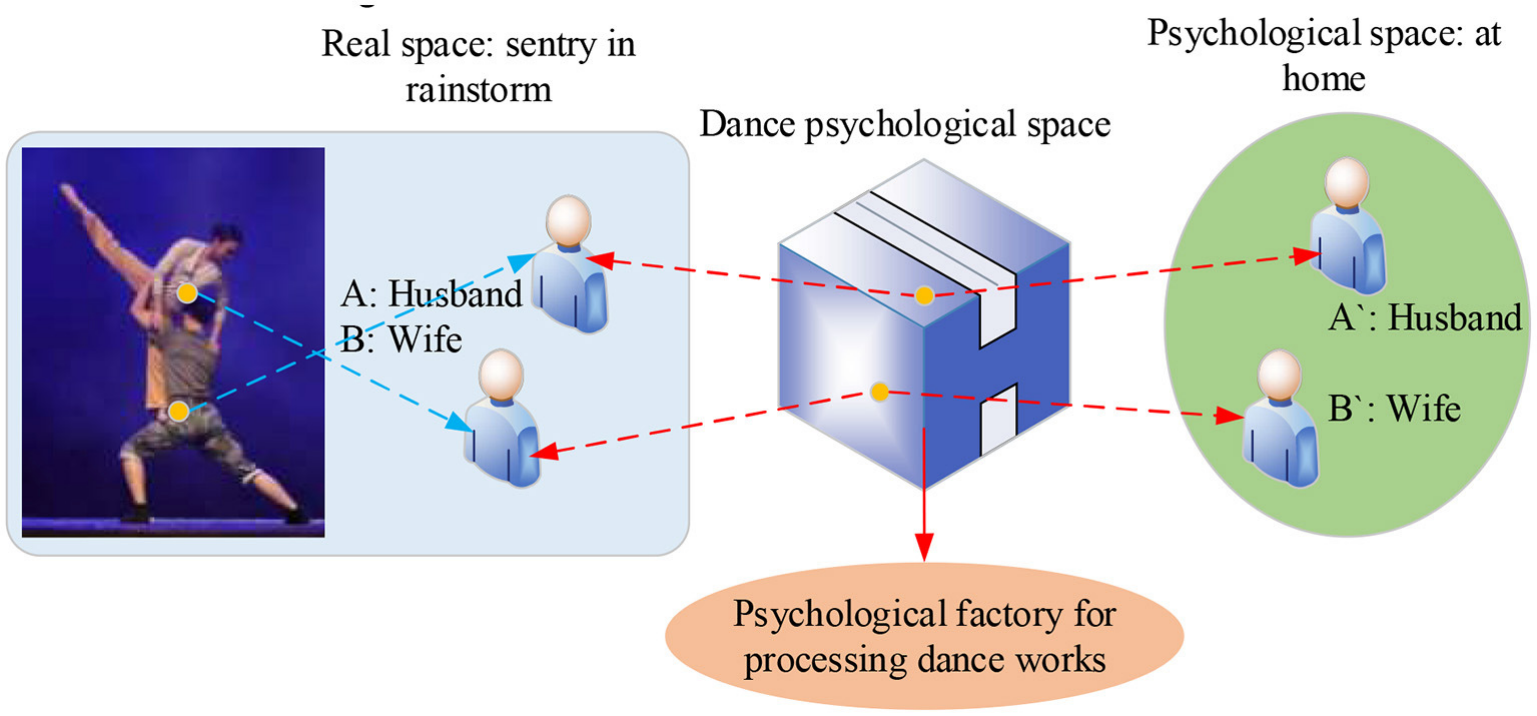
Schematic diagram of psychological construction of dance *A Couple Guarding A Sentry Box*.

From the perspective of art, the Psychological Construct is mainly affected by choreographers' experiences in real life. In the art of dance, to pursue a better theme, the artistic works must appeal to choreographers' thoughts and emotions through a vague mental state in the psychological space. For example, in the dance *A Couple Guarding A Sentry Box*, the second part involves a strong Psychological Construct by integrating real-life body language into dance moves. Thus, the dance has a solid real-life basis, reflecting the couple's life around the front line of fighting floods and disasters. Despite all the difficulties, the soldier's wife is brave and determined to support her husband: a People's Liberation Army (PLA) member, and share their joys and pains. Overall, the dance can well reflect ordinary people's lives and tendering emotions, thus infecting the audience. The two actors accurately convey the characters' psychology through aesthetic dance moves, such as modesty, heartache, helplessness, comfort, coquetry, marital love, and other real-life scenes. Meanwhile, the director has consolidated the characters' emotions into each move and embeds their psychological rhythm into the dance connection. The process of the Psychological Construct is demonstrated in Figure 2.

Figure 2 is the mapping diagram of Psychological Construct during the performance of the dance *A Couple Guarding A Sentry Box*. Obviously, the dancers have successfully expressed the characters' emotions and psychology through dance moves. Such is the process of Psychological Construct.

### Principle of Back Propagation Neural Network (BPNN)

**Figure 3:**
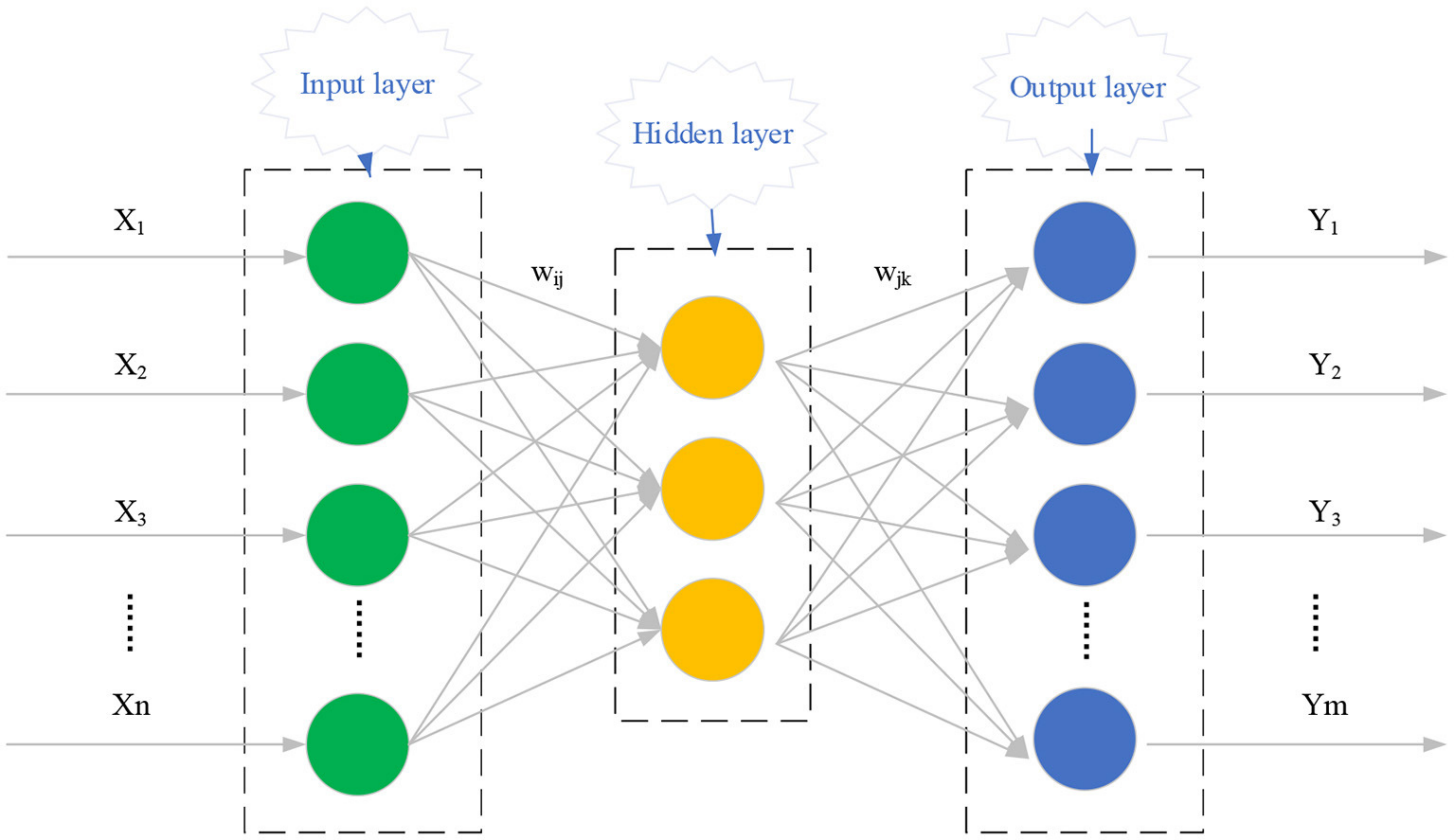
Three-layer ANN structure.

Based on the achievements of DL in human keypoint recognition, the Three-dimensional (3D) position coordinates of human key points are obtained in the image by analyzing RGB images. BPNN is a multilayer Artificial Neural Network (ANN), a new research direction in the DL field. It is introduced into a broader concept of Machine Learning (ML) to help realize true AI. In particular, AI aims to understand the essence of intelligence and fabricate an intelligent machine with human intelligence. It involves multiple disciplines, such as robotics, language recognition, Image Recognition (IR), Natural Language Processing (NLP), and Expert Systems (ES). BPNN features error back-propagation and signals forward transmission. Figure 3 sketches the structure of a three-layer ANN.

As detailed in Figure 3, generally, the ANN comprises three neuron layers: input layer, hidden layer, and output layer. The signal is fed into the input layer during the forward transmission, processed by the hidden layer, and outputted by the output layer. The lower-layer neurons' state is only affected by the state of neurons in the upper layer. Nevertheless, the error between the actual output and the expected result (if large enough) will be back-propagated to adjust the network threshold and weight. Such error back-propagation process will repeat itself until the predicted output of BPNN approach the expected output.

### Biped Humanoid Robot-Imagined Choreography Model

**Figure 4:**
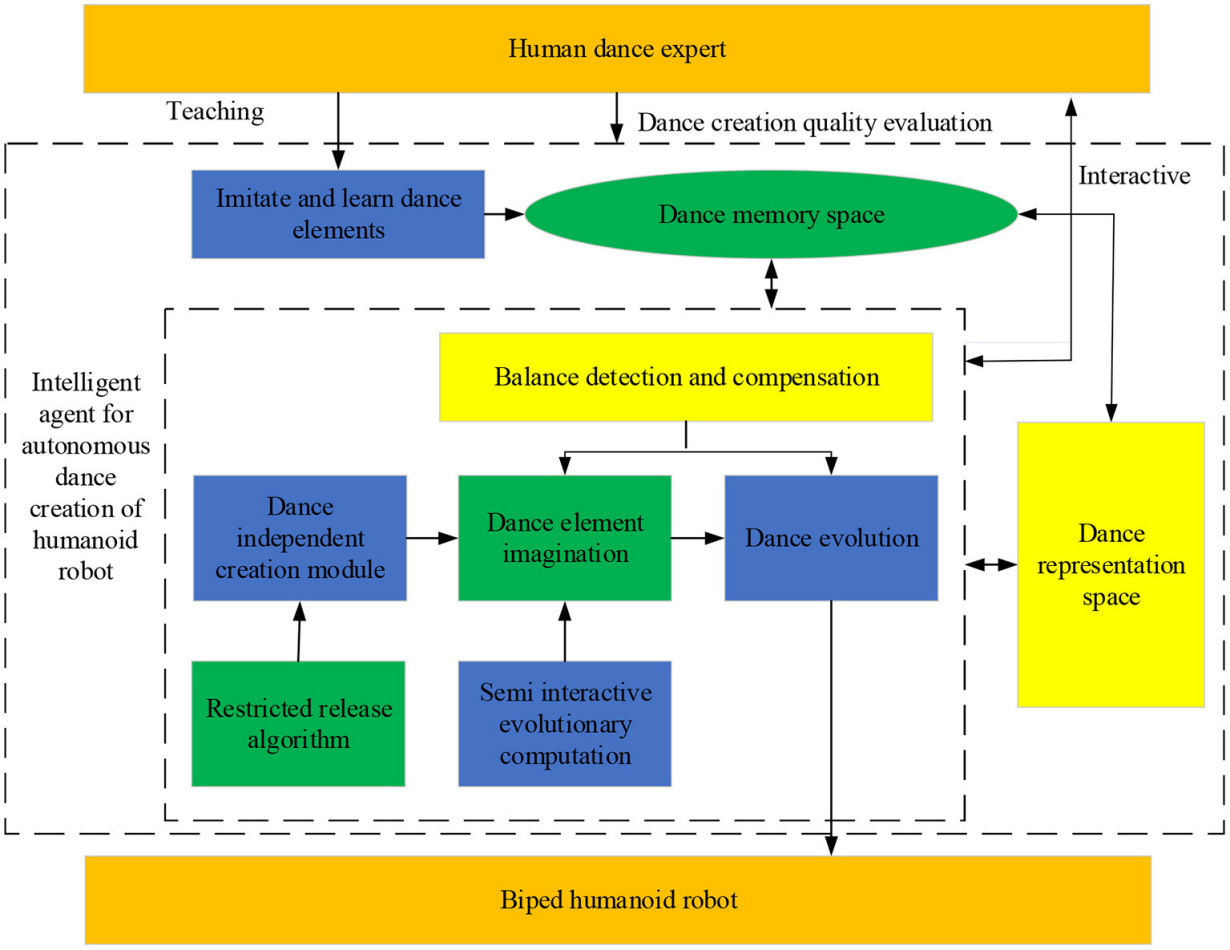
Framework of humanoid biped robot-imagined choreography model.

In addition to adopting the idea of choreography, choreographers also follow the following creative rules. (1) Learn and master the core elements of some dance forms, such as the shape and position of hand and feet shape, position, and speed. New elements lay the foundation of spontaneous imagination. (2) All these elements are used as the basic dance, and the dance movement is innovated, conforming to human body aesthetics and design. (3) These dance movements are associated together with different combinations, fabricating an intact dance work. Based on the above BPNN research and the objective rules of human choreography, the biped robot can imitate human thinking when choreographing the independent dance. The specific flow is as follows. Learning → Memorizing → Imagining → Processing → Combining. In order to efficiently complete choreography independent of human thought on the biped robot platform, the model is implemented, as given in Figure 4.

The joint angle is given as the action command of the robot. The robot's inverse kinematics calculation and forward kinematics calculation involves the mutual conversion between the joint coordinates and the joint angle. Moreover, the angle must be adjusted in multiple steps. This complex angle adjustment is unnecessary when the robot cannot imitate some delicate motions due to the freedom and mechanical structure constraints.

Figure 4 displays the choreographic process by a humanoid robot. “Dance space representation” (Martinez Damia et al., 2021) represents all the concepts adopted, including related concepts of joints and limbs on top of the traditional dance-related concepts, such as dance elements and posture. Moreover, the “memory dance space” stores (memorizes) all objects. These objects incorporate the robot's learned dance elements and imagined new dance elements and postures, which can be exported as executive files or relational databases. From the operation process of the model, the robot first watches the basic dance elements displayed by dance experts and then learns these basic dance elements through imagination. Based on this, it expands the new dance elements through its independent imagination, which will be used as the basis for the “imagination” of the next dance. At the same time, machine learning is introduced to enable the robot to appreciate aesthetic dance moves as humans so that it realizes the initial imaginary dynamic dance posture. The robot randomly connects the dance elements of various body parts with the dance posture in line with human aesthetics. Finally, the dance posture corresponding to human aesthetics is associated with different combinations. Consequently, a robot's independently choreographed dance is created and mapped to the physical platform of a biped robot. Further, three methods are adopted to smooth the choreography process of humanoid robots: semi-interactive evolutionary calculation, release-constrained algorithm, and balance detection and balance compensation (Liu, 2020).

The dance representation space of the humanoid robot can be represented by HRDES = {K, B, L, D, DA, DN, DC}. The meaning and expression of each part are listed in Table 1.

**Table 1:** Humanoid robot dance representation space: HRDES.

**Category**	**K**	**B**	**L**	**D**
Meaning	Joint set	Domain set	Limb set	Constraint set
Representation	*K* = {*K*_1_, *K*_2_, ⋯, *K*_|*K*|_}	*B* = {*B*_1_, *B*_2_, ⋯, *B*_|*K*|_}	*L* = {*L*_1_, *L*_2_, ⋯, *L*_|*L*|_}	*D* = {*D*_1_, *D*_2_, ⋯, *D*_|*L*|_}
Other	*K*_*i*_ represents the *i*th joint of the humanoid robot.	*B*_*i*_ represents the domain corresponding to the *i*th joint.	*L*_*i*_ represents the *i*th limb of the humanoid robot.	*D*_*i*_ represents the constraint on the *i*th limb, where *D*_*i*_∈{0, 1}.
Category	DA	DN	DC	
Meaning	Dance Collection	Dance posture	Dance routine	
Representation	*DA*_*i*_ = {*V*_*i*, 1_, *V*_*i*, 2_, ⋯, *C*_*i*, |_*L*__*i*_|_}	*DN* = {*DN*_1_, *DN*_2_, ⋯, *DN*_|*L*|_, *B*}	*DC* = {*DC*_1_, *DC*_2_, ⋯, *DC*_*Y*_}	
Other	*DA*_*i*_ means the limb *L*_*i*_'s dance elements, and *V*_*i, t*_ is the value of limb *V*_*i, t*_ at the *i*th joint.	Dance posture includes all dance elements of humanoid robot members and their aesthetic evaluation value B	A robot dance routine consists of a dance sequence with a length of Y.	

### Learning Basic Dance Elements and Imaging New Elements

Before independent choreography, the robot should first determine what dance postures to choreograph (Kashyap et al., 2020a). By defining dance postures, the robot can retain basic dance features of human dance moves (García and Diogo, 2020). HRDES indicates that dance elements are attached to limbs or expressed through specific dance postures, such as limb movement. Therefore, human limb movements must be manually classified to construct the limb movement set (*L*) before independent robot choreography. Then, the robot will learn all the dance elements demonstrated by human expert dancers alongside various dance postures. Different dance elements and postures combinations will be generated and stored in the robot dance memory space.

**Figure 5:**
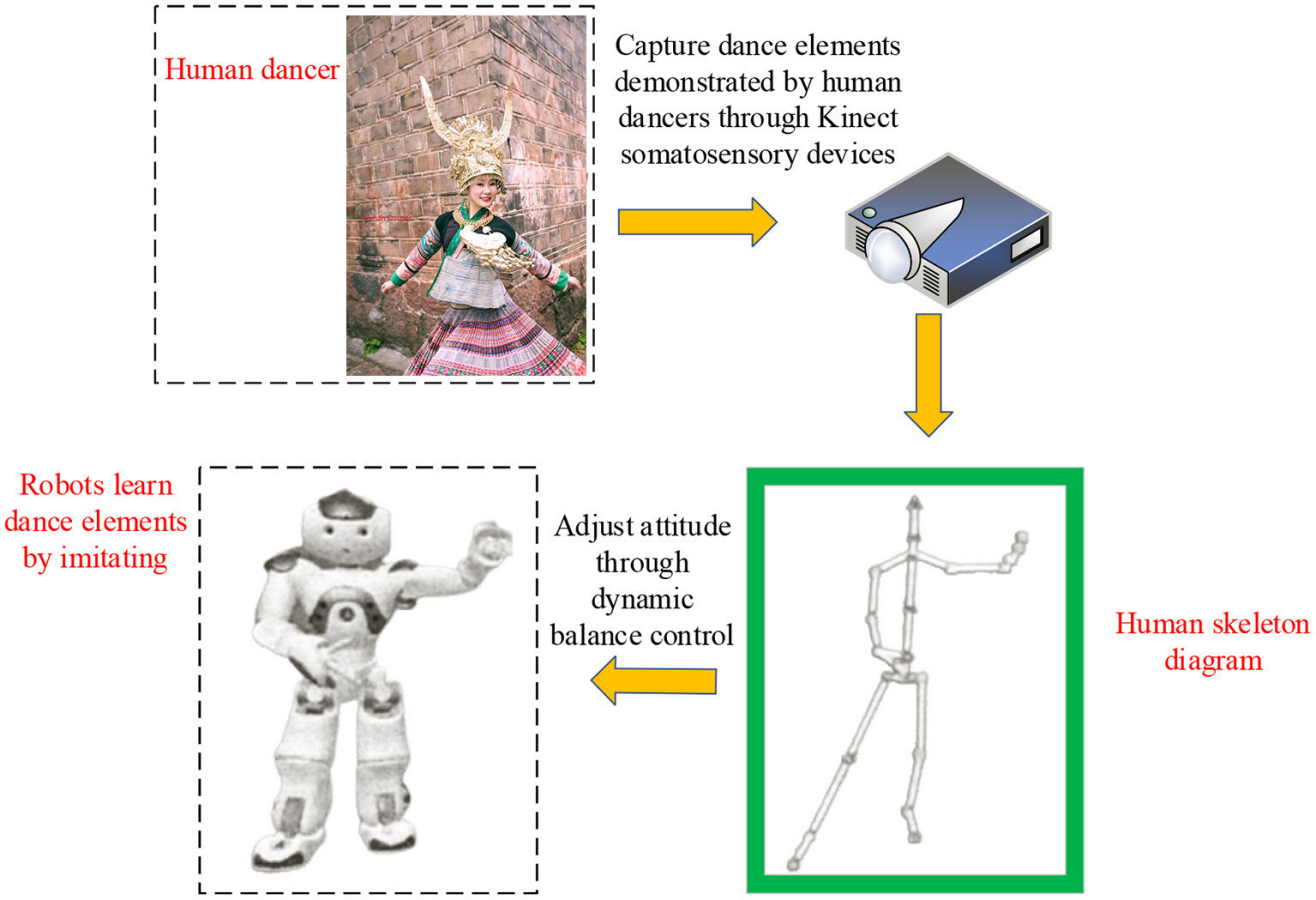
Basic dance elements imitation based on PSI.

Next, the simulation learning will be implemented based on Posture Similarity Index (PSI), referring to literature (Saqlain et al., 2020). The robot's human dance elements learning flow is illuminated in Figure 5.

**Figure 6:**
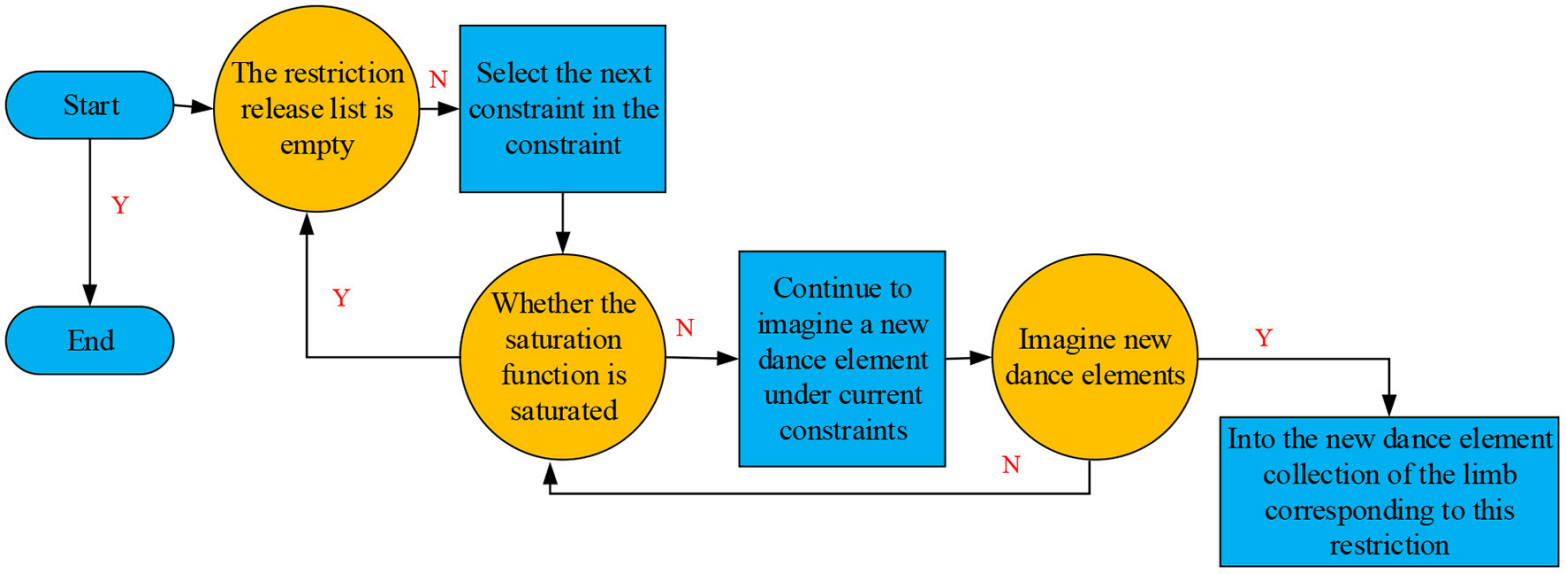
Algorithm flow of imagining new dance elements using the release-constrained algorithm.

According to the learned dance elements, the robot's newly imagined elements align with the general motion perception system, so the robot-imagined choreography based on the release-constrained algorithm is reasonable (Kashyap et al., 2020b). The release-constrained algorithm first clarifies the direction of constraint; each robot member without constraints is imposed with constraints to retain the human dance features. The constraints on robots maintain the dance elements' original features and do not allow “imagination” expansion. Figure 6 depicts the schematic diagram of the humanoid robot developing new dance elements “Imagination using the release-constrained algorithm.

As described in Figure 6, the corresponding robots can spontaneously “Imagine” new dance elements once the constraints are released. When all the constraints are released successively, all robot limbs without constraints are free to “image,” thus innovating new dance elements while maintaining original dance features.

Every imagined dance element on limb *L*_*i*_ must be verified. The dance element *DA*_*i*_ in dance representation space is defined as a vector, and an n-dimensional space *R* is composed of n components. Thus, the dance element *DA*_*i*_ is the next point of space *R*. Then, to determine *DA*_*i*_, a neighborhood d_i_ is first decided on the limb *L*_*i*_, a parameter to balance the number of imagined new dance elements and the original dance elements. It is a positive integer ϵ [1, MAX_i_]. Suppose Dmin[1owerbound, upperbound] is the smallest field in the corresponding fieldset of all joints of limb *L*_*i*_. In that case, MAX_i_ can be calculated by Equation (1):


(1) $$\begin{array}{r} MAX_{i}\;=\;\left[\frac{D_{\min}\cdot upperbpund\;-\;D_{\min}\cdot lowerbpund\;+\;1}{2}\right] \end{array}$$

The larger the neighborhood is, the fewer dance elements are in *R*, and the greater the difference between dance elements is, and vice versa. Therefore, an appropriate neighborhood *d*_*i*_ should be determined. All the neighborhood points (*d*_*i*_) are similar, yet each is a unique dance element point. In *R*, two spatially distanced points and no less than 2*d*_*i*_ are deemed distinguishable dance elements. In Equations (2) and (3), *DA*_*i, u*_ and *DA*_*i, v*_ represent two dance elements on the same limb *L*_*i*_, their PSI [*S*(*DA*_*i, u*_, *DA*_*i, v*_)] is calculated by Equation (2), and Equation (3) judge whether the two elements are identical.


(2) $$\begin{array}{r} S\left(DA_{i,u},DA_{i,v}\right)\;=\;EuclideanDistance\left(DA_{i,u},DA_{i,v}\right) \end{array}$$

(3) $$Same(DA_{i,u},DA_{i,v})\;=\;\{\begin{array}{c} \begin{array}{c} True\;if\;S\left(DA_{i,u},DA_{i,v}\right)\;<\;2d_{i} \end{array}\\ \begin{array}{c} False\;if\;S\left(DA_{i,u},DA_{i,v}\right)\;\geq \;2d_{i} \end{array} \end{array}$$

Next, a new dance element imagination algorithm is given based on the change of single joint value on limb *L*_*i*_. As shown in Algorithm 1:

**Algorithm 1:** Algorithm flow of the new dance element imagination based on the change of single joint value.

For (Select each limb *L_*i*_* in turn from the limb set L)
*Li* = {*J*_*i*, 1_, *J*_*i*, 2_, ⋯ , *J*_*i*, |_*L*__*i*_|_}
If D_i_= 0 Then
For (In limb *L*_*i*_, select each learned basic dance element *DA_*i*_* in order)
*DA_*i*_* = (*V*_*i*, 1_, *V*_*i*, 2_, …, *V*_*i*, |_*L*__*i*_ |_)
{
If not repeat(*DA_*i*_*) Then
The learned basic dance element *DA_*i*_* is added to *W*;
For (Select each joint *K*_*i, k*_ in order on the limb *L_*i*_*)
*k*∈[1, |*L*_*i*_|]
{
For (Select each value V^′^ on the domain *D_*k*_* [lowerbound, upperbound] corresponding to joint *K_*i, k*_*, with a step size of 2d)
{
Imagine and choreograph a new dance element $DAi^{\prime }(V_{i,1},\;V_{i,2},\cdots ,\;V_{i,k-1},V^{\prime },\;V_{i,k+1},\ldots ,V_{i,\left|L_{i}\right|})$ on the limb L_i_;
If not repeat(*DA_*i*_*') Then
The imaginary new dance element *DA_*i*_*' is added to *W*;
}
}
If full Then
Ends the imagination on the limb *L_*i*_* and jumps to the outer layer of the cycle to select the next limb;
}

### Model Experiment and Analysis

Subsequently, this section takes Tibetan tap dance as an experimental subject for independent humanoid robot-imagined choreography. The Tibetan tap dance features regular knee swings and footsteps (Hannah and Red, 2020). In particular, the experimental robot selects the most popular bipedal robot in AI research: the NAO robot (Robaczewski et al., 2021), which also sees wide applications in teaching, smart home, entertainment, and competition. Because of the superior processing mechanism for joint and body balance, NAO can present various body postures and is an ideal platform for studying biped robot dance. For model implementation convenience, NAO is used in the simulation environment. At the same time, Webots (Sun et al., 2022) are employed, a powerful environment development software for mobile robot modeling, programming, and simulation.

The experimental simulation environment is based on a personal computer. The software environment adopts Windows 7.0 Operating System (OS) and Webots 7.0 4.1 environments. The “memory dance space” is the Windows file system, where all instantiated objects are stored. The visual C++ platform is chosen for coding (Noor and Saad, 2021). In Webots, the hardware is configured with the robot NAO and the computer, which are used to visualize various objects (dance elements, dance posture, and overall dances).

**Figure 7:**
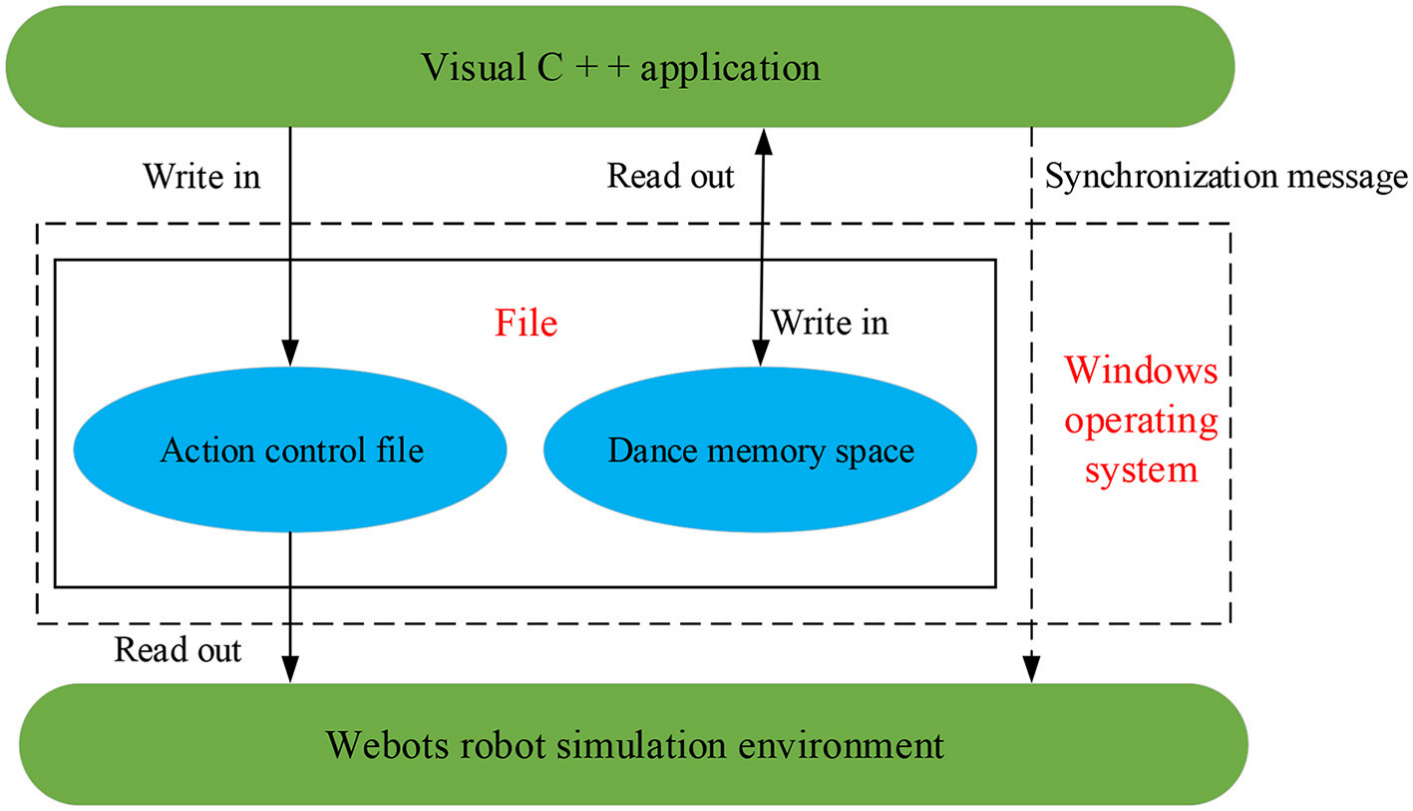
Relationship among Windows, Webots, and Visual C++.

The files in Windows OS, Webots environment, and Visual C++ are closely related, as manifested in Figure 7. There is one-way data transmission between Webots and Visual C++. Visual C++ generates the shared action control file to simulate NAO. Webots read the shared action files and display dance objects (dance posture, dance elements, and the overall dance) on the simulated NAO. The Windows Messages can synchronize the visual application Webots and C++. Because the dance memory space is implemented on Windows, Visual C++ must load and compile the whole implementation process of the dance object model. Thus, Visual C++ will repeatedly access the files in the dance memory space.

The proposed intelligent robot-imagined independent choreography model needs to learn the basic dance elements from dance professionals. This study chooses the Tibetan tap dance for the humanoid robot to imitate and learn. Human dancers participate in dance posture evaluation through semi-interactive evolutionary computing. Then, 10 ethnic dance professionals, who never used biped robots, are invited from Dance University to evaluate and analyze the robot-imagined dance postures. Nevertheless, they have rich experience in stage performance, choreography, and teaching. Everyone has a high aesthetic evaluation ability and aesthetic perception. To improve the dance posture evaluation effectiveness, these dance professionals are informed in advance that the dance style of the model robot is Chinese Tibetan tap dance.

First, the dance representation space of the humanoid robot must be instantiated (Slot et al., 2020). According to the NAO technical manual, NAO is designed with 26 joints with corresponding motion ranges to determine groups K and B in the dance representation space. Because of the characteristics of tap dancing and the physical structure limitations of the NAO robot, the whole robot body is divided into three parts, namely, *L* = *{L*_1_*, L*_2_*, L*_3_), with the constraints *D* = {*D*_1_, *D*_2_, *D*_3_), as unfolded in Table 2. *L*_3_ (lower-body) plays an essential role in body balance and reflects the features of Tibetan tap dance. Thus, constraints are imposed on limb *L*_3_ to maintain the dance features, and *D*_3_ = l. In other words, it is not allowed to imagine dance postures on *L*_3_ limbs. In Tibetan tap dance, human dancers often keep their hands relaxed. Accordingly, the NAO robot's left arm and right arm are modeled as the upper body *L*_2_ (Upper-body), not independently. Compared with *L*_3_, *L*_2_, and *L*_*l*_ (head) are more flexible, so *D*_1_ = 0 and *D*_2_ = 0. Hence, *L*_2_ and *L*_*l*_ can be imagined with new dance elements based on the learned dance elements on *L*_3_. The joint set corresponding to limb *L*_1_ is {HeadYaw, HeadPitch}. The joint set corresponding to limb *L*_2_ is {LShoulderPitch, LShoulderRoll, LEbowYaw, LElbowRoll, LWristYaw, LHand, RShoulderPitch, RShoulderRoll, RElbowYaw, RElbowRoll, RWristYaw, RHand}. The joint set corresponding to limb *L*_3_ is {LHipYawPitch, LHipRoll, LHipPitch, LKneePitch, LAnkiePitch, LAnkleRoll, RHipYawPitch, RHipRoll, RHipPitch, RKneePitch, RAnklePitch, RAnkleRoll}.

**Table 2:** Comparison between the proposed model and IEC.

**Models**	**Evaluation method**	**Final evolutionary outcome**	**Human load in HCI**	**Is there a leg dance?**	**Can the method retain the original features of dance?**
Literature (Li et al., 2020) choreography model	Human evaluation	Optimal solutions (less)	Heavier	No	Cannot
The proposed model	Human + machine evaluation	Better solutions (more)	Lighter	Yes	Can

### Instantiation of Dancing Robot HCI Interface

**Figure 8:**
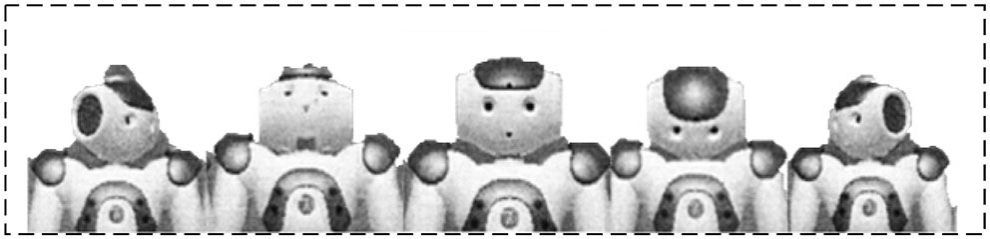
Basic dance elements imitated on upper body *L*_2_ (upper-body).

**Figure 9:**
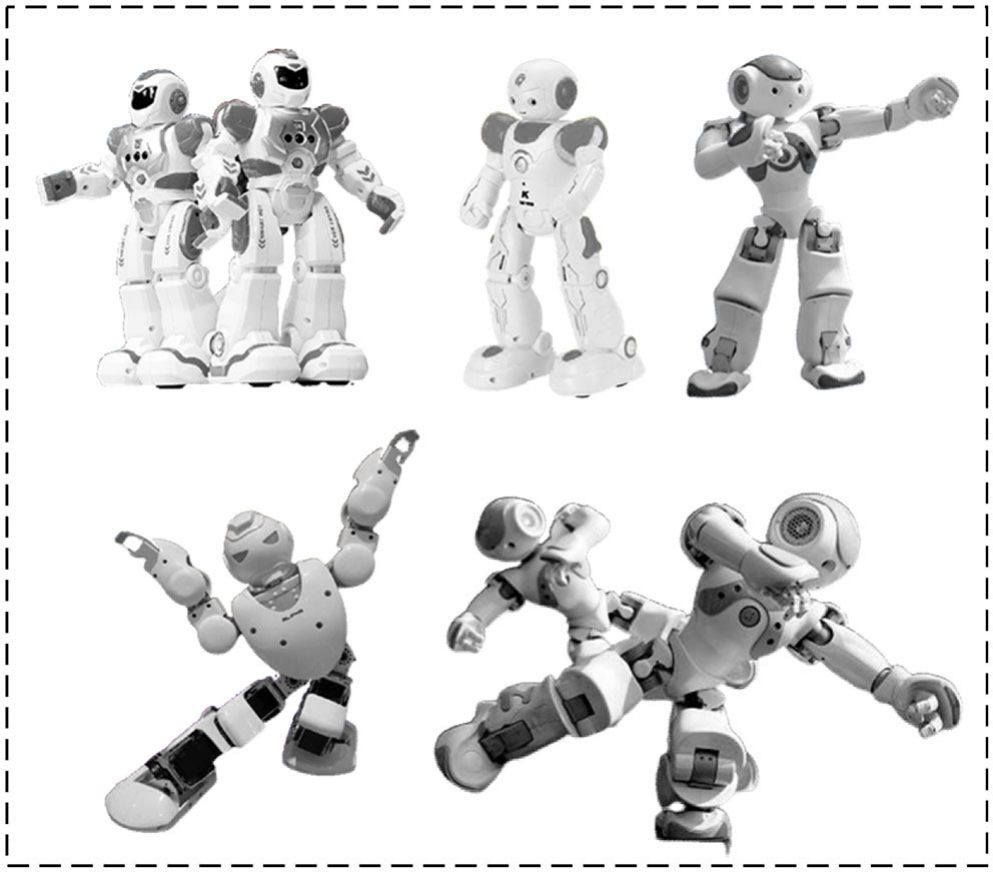
Basic dance elements imitated on limb *L*_1_ (head).

**Figure 10:**
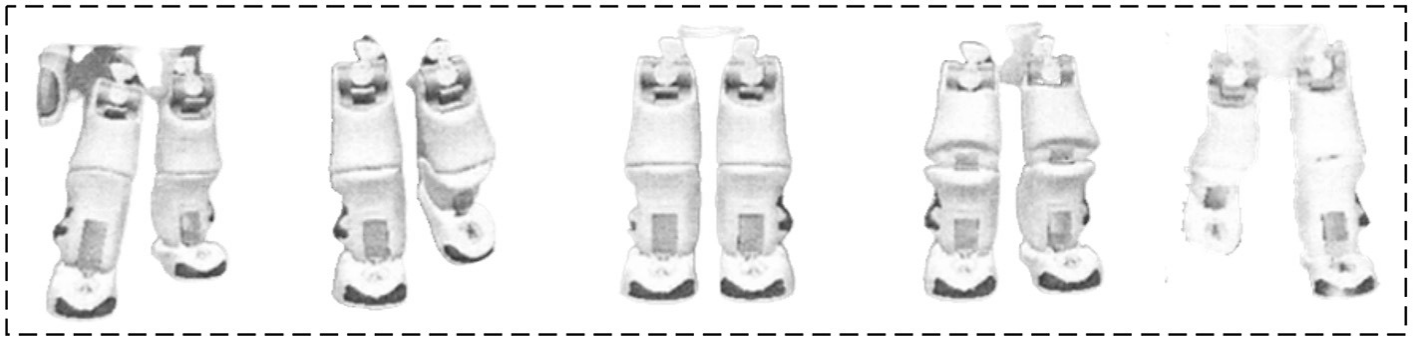
Basic dance elements imitated on lower body *L*_3_ (lower body).

Based on the dance representation space of the instantiated humanoid robot, the PSI-based simulation learning method is adopted to learn the basic dance elements of human dancers. Thus, the robot's basic dance elements on three limbs (*L*_1_, *L*_2_, *L*_3_) are collected and stored in the dance space. Specifically, seven basic dance elements are learned on the limb *L*_2_, as given in Figure 8. Five basic dance elements are learned on the limb *L*_1_, as shown in Figure 9. Another five basic dance elements have been learned on limb *L*_3_, as depicted in Figure 10. Additionally, to enable the robot to imagine more new dance elements on limbs *L*_1_ and *L*_2_, the parameters are set as *d*_1_ = 1 and *d*_2_ = 1.

**Figure 11:**
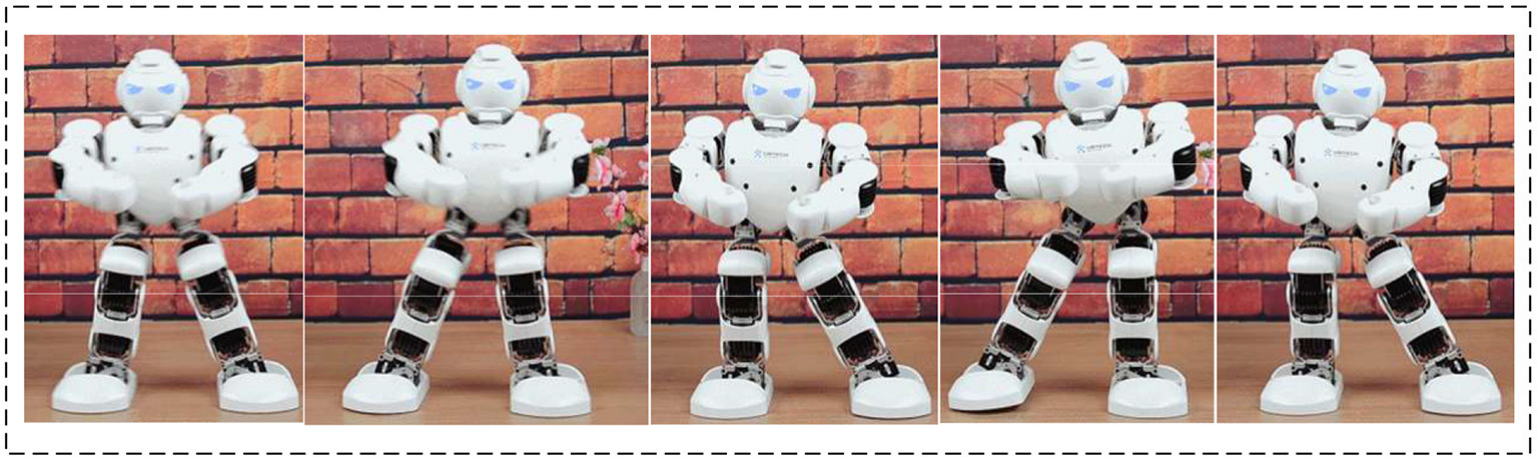
An “Excellent” dance posture imagined by the humanoid robot.

Then, multiple “excellent” dance postures are randomly picked out and are combined with different combinations for the robot to image new dance elements. Such is the whole process of independent robot-imagined choreography. Figure 11 charts one dance posture imagined by the proposed robot-imagined choreography model.

## Results and Discussion

### Analysis and Evaluation of New Elements of Imaginative Dance

The experimental part mainly studies a kind of Tibetan tap dance. The proposed robot-imagined choreography model uses the release-constrained algorithm on *L*_1_ and *L*_2_. Altogether, 986 and 23,421 new dance elements are imagined on *L*_1_ and *L*_2_.

**Figure 12:**
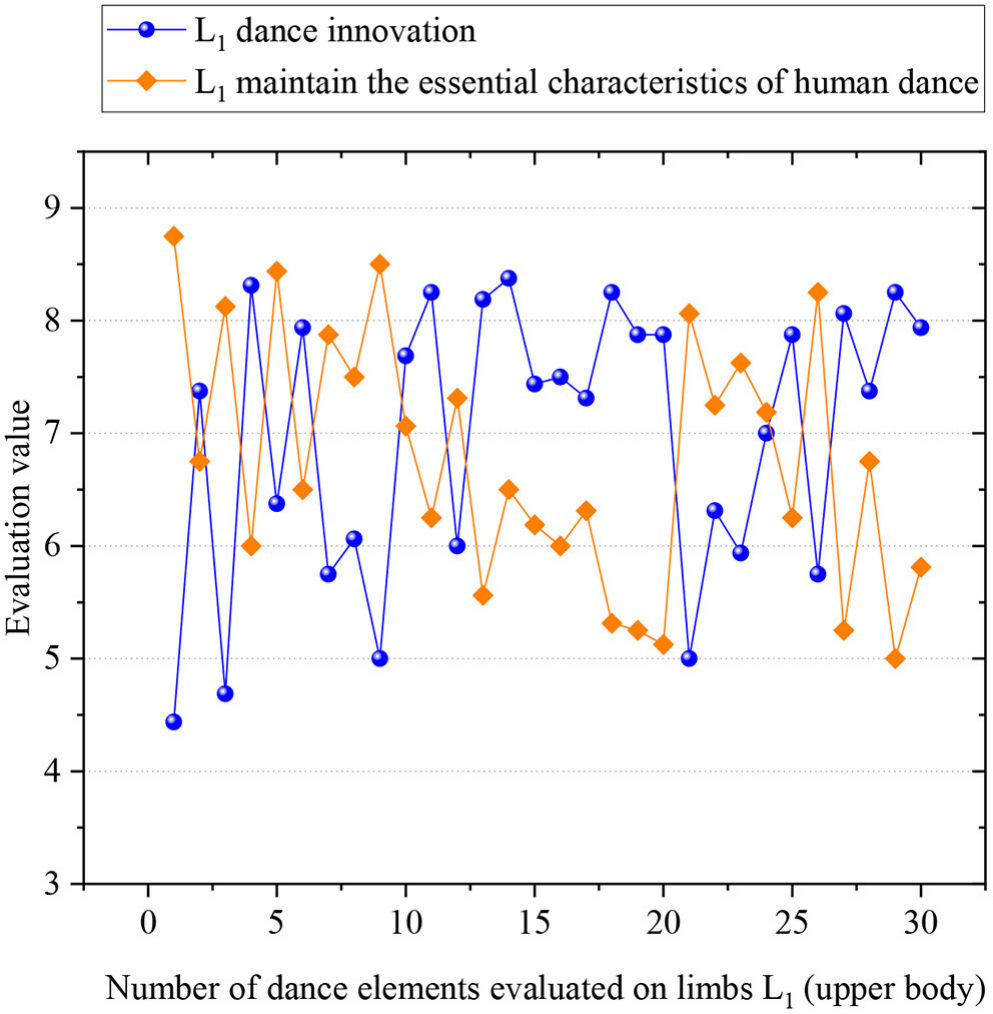
Evaluation scores of robot-imagined 30 new dance elements on *L*_1_.

**Figure 13:**
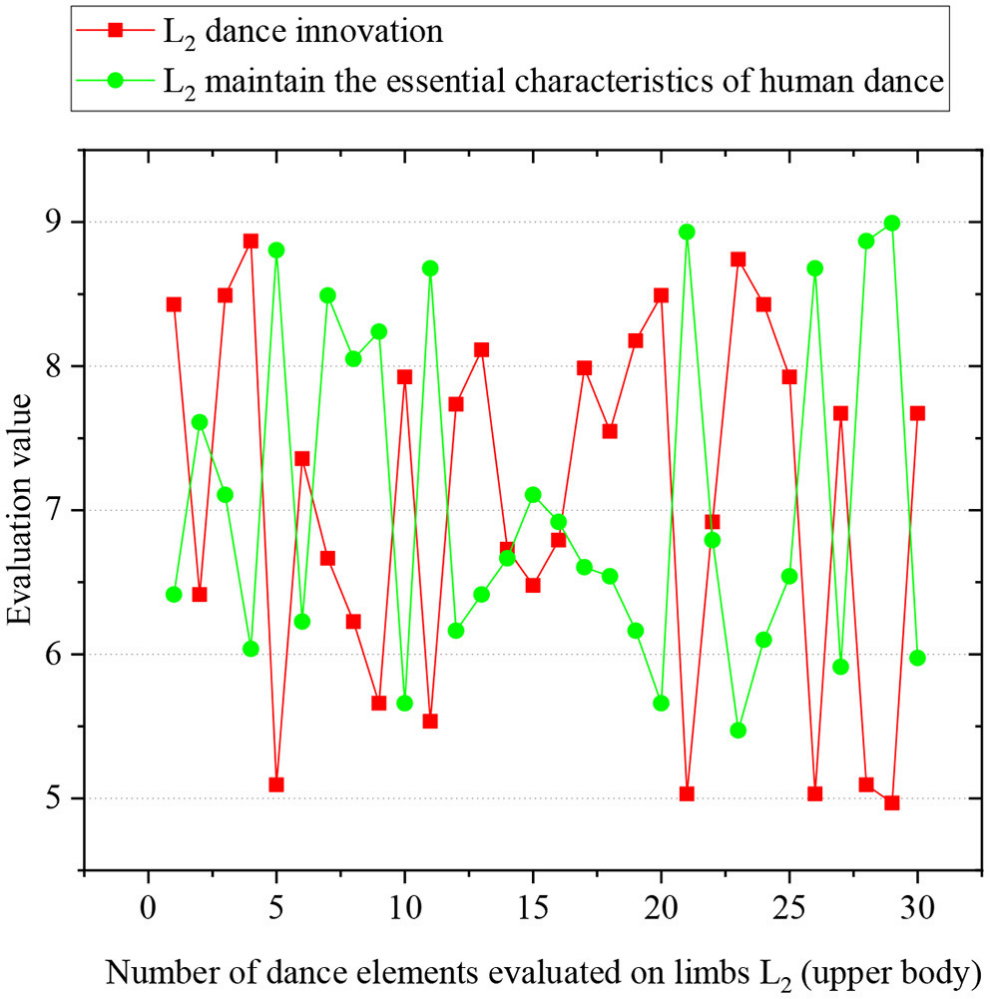
Evaluation scores of robot-imagined 30 new dance elements on *L*_2_.

Then, 30 new dance elements on *L*_2_ and 30 on *L*_1_ are randomly selected for model evaluation. Ten expert dancers are invited to evaluate the innovation and basic features of the robot-imagined dance postures. Each expert is told to evaluate every dance element and score an integer between (Li, 2020; Martinez Damia et al., 2021) (1—the worst, and 10—the best). When all experts finish scoring, the lowest and highest scores are removed to avoid the negative impact of extreme data. The remaining scores are averaged as the final score of the dance element. Figures 12, 13 illustrate the evaluation results of robot-imagined new dance elements on *L*_2_ and *L*_1_. The *y*-axis represents the evaluated score of dance elements, and the *x*-axis is the number of dance elements. The two lines stand for the evaluation sores of dance innovation and retention of the basic dance features, respectively.

After calculation, the average basic feature retention score of 30 new elements on the *L*_2_ is 7.73, and the average innovation score is 7.40. Those scores on *L*_1_ are 7.29 and 7.64, respectively. In summary, the proposed robot-imagined choreography model can produce innovative dance elements while maintaining the basic dance features. Also, it comprehensively considers the contradiction between the basic dance features and innovation.

### Comparison of Two Choreography Methods

Table 2 compares the Interactive Evolutionary Computing (IEC) method in literature (Li et al., 2020) with the proposed bipedal-robot-imagined choreography model.

In the proposed choreography model, the body of the robot NAO is divided into several members according to the features of the selected dance. Then, dance elements are extracted from different members. At the same time, any dance element, whether imitated or imagined, always retains the essential features of human dance. Therefore, the combined dance posture still retains the essential features of human dance. By comparison, the robot dance method based on IEC does not consider how to maintain the basic features of human dance.

### Influence of the Dancing Robot on Choreography

As far as dance itself is concerned, dancing robots can broaden the expression of the art of dance. Interestingly, dance uses body language to express emotion and intention, with both artistic and decorative utilities (Lin et al., 2022). Currently, robots might not dance as flexibly and aesthetically as human dancers. Nevertheless, thanks to robots' increasingly intelligent humanoid features, robot-expressed dance will soon cause emotional resonance with the audience and influence the audiences' emotional expression. In the process of robot-expressed dance, factors affecting the audience's judgment also have particular research significance (Rifajar and Abdul, 2021). The sense of rhythm, the coherence of movement, and the symmetry of movement will affect the effect of dance, which requires reflection and research on the dance art itself. In addition to dancers' personalities, robot-expressed dance can also provide a research platform for analyzing and researching dance as an art (Li et al., 2020). Further, robot-expressed dance also improves people's understanding of dance itself. Robot-imagined choreography can stimulate authentic human choreography and enhance efficiency for professional dancers. Last, from a commercial point of view, the entertainment of dancing robots and their interaction with people make it a vital R&D field in the field of home service robots. During the HCI with dancing robots, people can be participants or evaluators (Peng et al., 2021). With human participation, the robot can imitate human action. At the same time, as a bystander, people can evaluate the robot's dance postures and feedback the information to the robot for self-improvement. Overall, as a popular form of performance, dance has the characteristics of rhythm, lyricism, and movement.

## Conclusion

Dance language has both common characteristics and special laws as a language phenomenon. Robot dance is an exciting research field that has attracted worldwide attention and research. First, this paper systematically reviews the research, summary, and classification model in the field of dancing robots. The innovation is the proposal of a new humanoid dancing robot model that can choreograph dance elements independently. The choreography refers to the human thinking mode. Specifically, it establishes a biped-humanoid robot to choreograph dance moves actively. Then, it invites human dance professionals to evaluate the robot-imagined dance posture to verify the feasibility and effect of the model. The 30 new dance elements imagined on the *L*_1_ get an average basic feature retention and innovation scores of 7.29 and 7.64, respectively. The results show that the proposed independent robot-imagined choreography model is superior to other literature methods and is feasible and innovative. However, there are still some deficiencies. The proposed model is based on the synchronous change of the value of one joint and the value of two joints. Future works are expected to design a new algorithm for dance element imagination based on the value change of multiple joints and maintain human dance's innovation and basic features.

## Data Availability Statement

The raw data supporting the conclusions of this article will be made available by the authors, without undue reservation.

## Ethics Statement

The studies involving human participants were reviewed and approved by Sultan Idris Education University Ethics Committee. The patients/participants provided their written informed consent to participate in this study. Written informed consent was obtained from the individual(s) for the publication of any potentially identifiable images or data included in this article.

## Author Contributions

The author confirms being the sole contributor of this work and has approved it for publication.

## Conflict of Interest

The author declares that the research was conducted in the absence of any commercial or financial relationships that could be construed as a potential conflict of interest.

## Publisher's Note

All claims expressed in this article are solely those of the authors and do not necessarily represent those of their affiliated organizations, or those of the publisher, the editors and the reviewers. Any product that may be evaluated in this article, or claim that may be made by its manufacturer, is not guaranteed or endorsed by the publisher.
